# Montelukast Inhibits HCoV-OC43 Infection as a Viral Inactivator

**DOI:** 10.3390/v14050861

**Published:** 2022-04-21

**Authors:** Yongkang Chen, Xiaohuan Wang, Huichun Shi, Peng Zou

**Affiliations:** Shanghai Public Health Clinical Center, Fudan University, Shanghai 201508, China; 21111010055@m.fudan.edu.cn (Y.C.); 18111010062@fudan.edu.cn (X.W.); 19211300001@fudan.edu.cn (H.S.)

**Keywords:** montelukast, HCoV-OC43, viral inactivator

## Abstract

Coronaviruses (CoVs) consist of a large group of RNA viruses causing various diseases in humans and in lots of animals. Human coronavirus (HCoV) OC43, the prototype of beta-coronavirus discovered in the 1960s, has been circulating in humans for long time, and infection with other emerging strains of beta-coronavirus (SARS-CoV, SARS-CoV-2, and MERS-CoV) can lead to severe illness and death. In this study, we found that montelukast, a leukotriene receptor antagonist, potently inhibited the infection of HCoV-OC43 in distinct cells in a dose- and time- dependent manner. Additionally, the results showed that montelukast induced release of HCoV-OC43 genomic RNA by disrupting the integrity of the viral lipid membrane, and irreversibly inhibited viral infection. Considering the similarity among HCoV-OC43, MERS-CoV, and SARS-CoV-2, it suggests that montelukast may be a potential candidate for the treatment of human beta-coronavirus infection.

## 1. Introduction

Coronaviruses (CoVs) are enveloped single-stranded RNA viruses. Their genome is positive-sense RNA of approximately 30 kb, which is the largest of all known RNA viruses. A wide range of animal species can be infected by CoVs, which may cause respiratory diseases with varying severity, including the common cold, bronchiolitis, and pneumonia [1,2,3]. CoVs are further classified into alpha, beta, gamma, and delta genera [4]. Seven CoVs were isolated from humans (HCoVs), including HCoV-OC43, HCoV-229E, HCoV-NL63, HCoV-HKU1, Middle East respiratory syndrome CoV (MERS-CoV), severe acute respiratory syndrome CoV (SARS-CoV), and SARS-CoV-2 [5].

HCoV-OC43 belongs to beta-coronavirus genus and was first isolated from a patient with respiratory disease in the 1960s [6]. In addition, this virus is recognized as the most prevalent human coronavirus in the world, and has the highest incidence in winter and spring [7]. Notably, HCoV-OC43 has been reported to be the most prevalent subtype of HCoVs and is considered as one of the most important pathogens responsible for the upper respiratory infections [8,9]. Several studies in the past years have also reported that infection with HCoV-OC43 leads to neurological diseases because of its neuroinvasive properties [10,11]. Similar to HCoV-OC43, three HCoVs with high pathogenicity (SARS-CoV, MERS-CoV, and SARS-CoV-2) are all subdivided into beta-coronavirus genus. Moreover, SARS-CoV-2 shares several functional properties with HCoV-OC43. The outbreak of SARS-CoV-2 has resulted in approximately 318 million confirmed cases and more than 5.5 million deaths as of 14 January 2022. There have been several COVID-19 vaccines and monoclonal antibodies approved for emergency use in the prevention and treatment of SARS-CoV-2 infection, while the emergence of SARS-CoV-2 variants, such as B.1.1.7, B.1.617.2, and B.1.1.529, with increased transmissibility and more resistance to vaccine sera or monoclonal antibodies, has seriously exacerbated the threat to the public health worldwide [12,13]. However, only few therapies with small molecule drugs targeting to beta-coronavirus directly have been approved for treatment with infection of SARS-CoV-2 currently, highlighting the urgent need to develop more treatments with potent efficacy [14]. 

It is widely known that it takes long time to develop a novel drug and to get approval for marketing after clinical trials. In this context, repurposing of Food and Drug Administration (FDA)-approved drugs may help to speed up the development of antiviral drugs against coronavirus, including both HCoV-OC43 and SARS-CoV-2, as rapid evaluation of the efficacy in vitro and in vivo is conceivable. HCoV-OC43, as the prototype of beta-coronavirus, is a useful alternative model for the highly pathogenic HCoVs in drug development, considering the limited access to biosafety level three containment facilities and its similarity with SARS-CoV, MERS-CoV, and SARS-CoV-2, which is evidenced by the fact that antiviral agents have been previously reported to suppress the replication of HCoV-OC43, and could be candidates for inhibiting other human beta-coronavirus infections, such as SARS-CoV-2 and MERS-CoV [15,16].

Montelukast, a leukotriene receptor antagonist safely used in asthma patients for many years, has the potential to inhibit SARS-CoV-2 and MERS-CoV infection [17,18,19] and has the ability to inhibit Zika virus infection by disrupting its integrity [20]. In this study, we found that montelukast could also effectively inhibit the infection of HCoV-OC43 by screening FDA-approved drugs. It showed that montelukast inhibited the infection of HCoV-OC43 in a dose- and time-dependent manner by inactivating HCoV-OC43 and blocking the viral entry. It is promising that montelukast may be a potential candidate of entry inhibitor for the treatment of human coronavirus infection, including HCoV-OC43, MERS-CoV, and SARS-CoV-2.

## 2. Materials and Methods

### 2.1. Cells and Viruses

Baby Hamster Kidney-21 (BHK-21) cells, rhabdomyosarcoma (RD) cells, HCT-8 human colorectal cancer cells, MRC-5 human normal diploid lung fibroblast cells, and HEK-293T cells were maintained in Dulbecco’s modified Eagle’s medium (DMEM; Biological Industries, Beit HaEmek, Israel) supplemented with 10% fetal bovine serum (FBS; Biological Industries, Beit HaEmek, Israel) at 37 °C and 5% CO_2_.

Human coronavirus OC43 strain (HCoV-OC43, VR-1558) was obtained from American Type Culture Collection (ATCC; Manassas, VA, USA) and propagated in RD cells. The viral titer was determined on BHK-21 cells.

### 2.2. Compounds and Antibody

Montelukast sodium (Mon) and chloroquine phosphate were bought from Sigma-Aldrich (St. Louis, MO, USA). Curcumin and digitonin were purchased from MedChemExpress (Monmouth Junction, NJ, USA) and Targetmol (Wellesley Hills, MA, USA), respectively. All compounds were dissolved in dimethyl sulfoxide (DMSO) and stored at −20 °C except chloroquine phosphate, which was dissolved in sterilized water. The mouse anti-HCoV-OC43 nucleoprotein monoclonal antibody (mAb) was purchased from Millipore (Temecula, CA, USA).

### 2.3. Plaque Assay

The titer of infectious HCoV-OC43 particles was determined by plaque assay performed on BHK-21 cells, as described before with minor modifications [21]. Briefly, BHK-21 cells were seeded in cell culture plates and incubated overnight to a confluent monolayer, then virus or sample (mixture of virus and compound) was added to cells. At 2 h post-incubating, the supernatant was removed from BHK-21 cells and covered with an overlay of DMEM, containing 2% FBS and 0.6% low-melting-point (LMP) agarose (Promega, Madison, WI, USA). The plates were further incubated at 33 °C for about 4 days, then plaques were visualized after fixing with 4% formaldehyde and staining with 1% crystal violet and counted using plaque forming unit (PFU).

### 2.4. Cell Viability Assays

RD, HCT-8, MRC-5, or HEK-293T cells (2 × 10^4^/well) were seeded into 96-well cell culture plates and grew overnight at 37 °C. Subsequently, cells were treated with different concentrations of montelukast, serially diluted from 160 to 1.2 µM in DMEM with 2% FBS. After 48 h incubation at 33 °C, cell viability was detected by the Cell Counting Kit-8 (CCK-8, Dojindo, Japan) according to the manufacturer’s instructions as previously described [20,22]. Then the absorbance at 450 nm wavelength was measured by the iMark TM microplate reader (Bio-Rad, Hercules, CA, USA).

Propidium iodide (PI) staining of RD cells were performed as described before with minor modifications [23,24]. RD cells were seeded in 24-well cell culture plates and incubated overnight at 37 °C. Cells were treated with montelukast (5, 10, and 20 µM) or digitonin (5 and 10 µM) respectively for 2 or 6 h at 37 °C. After removing the supernatant and washing cells twice with fresh DMEM, 10 µg/mL of PI in DMEM was supplemented for 20 min incubation. Then, the cells were observed for PI staining with a fluorescence microscope.

### 2.5. Antiviral Assays

BHK-21 cells were seeded into cell culture plates and grew overnight to a confluent monolayer. 100 PFU of HCoV-OC43 was mixed with decreasing concentrations of montelukast ranging from 5 to 0.3125 µM and incubated 1 h at room temperature, then added to each well of plates. After incubation for 2 h at 33 °C, the plaque assay was performed and analyzed as described above. The inhibition of HCoV-OC43 infection by the montelukast was calculated and the 50% inhibitory concentration (IC50) value was determined by using the software CalcuSyn kindly provided by Dr. T.C. Chou [25].

RD, HCT-8 or MRC-5 cells (2 × 10^5^) were infected with 2 × 10^4^ or 2 × 10^5^ PFU of HCoV-OC43 after viruses were treated with or without 10 µM montelukast for 1 h at 33 °C. Then, the inoculum was removed from cells after 2 h incubation at 33 °C and fresh medium, containing 2% FBS and supplemented with or without 10 µM montelukast, was added. Then, the supernatant was collected at 48 or 72 h after infection, and the infectious HCoV-OC43 particles were determined by plaque formation assay as described above.

The inhibition of montelukast against HCoV-OC43 in multiple rounds of infection was also analyzed in the HCT-8 cells infected with viruses pretreated by 10 µM of montelukast for 1 h at 33 °C. At the indicated time points post infection, the titers of HCoV-OC43 in the supernatant were determined by plaque assay using BHK-21 cells as described above.

To determine the time dependence for inactivation of montelukast on HCoV-OC43, 100 PFU of HCoV-OC43 was incubated with different concentrations of montelukast (10, 5 and 2.5 µM) or without montelukast for the indicated times (15, 30, 60, and 120 min), and the plaque assay was immediately determined on BHK-21 cells as described above.

### 2.6. Flow Cytometry

HCoV-OC43 (2 × 10^4^ PFU) was treated with serially diluted montelukast for 1 h at 33 °C. Then, the mixture was added to RD cells or MRC-5 cells (2 × 10^5^) and incubated for 2 h at 33 °C. Subsequently, the inoculum was removed and DMEM containing serially diluted montelukast and 2% FBS was supplemented. At 24 or 48 h post-infection, RD or MRC-5 cells were trypsinized by Trypsin/EDTA (Biological Industries, Beit HaEmek, Israel), respectively, then fixed and permeabilized with the BD Fixation/Permeabilization Kit (BD Biosciences, San Jose, CA, USA) and stained with the mouse anti-HCoV-OC43 nucleoprotein mAb diluted 1:1000 and a rabbit anti-mouse IgG coupled to fluorescein isothiocyanate (FITC) (DAKO, Glostrup, Denmark), diluted 1:400. Flow cytometry was carried out in a FACSCalibur cytometer (BD Biosciences) and analyzed by FlowJo software version 10 (BD Biosciences, San Jose, CA, USA).

### 2.7. Time-of-Addition Assay

The time-of-addition experiment for montelukast was performed as previously described to determine at which stage the drug exhibited inhibitory efficacy [20,26,27]. BHK-21 cells seeded in 6-well plates overnight to a confluent monolayer were infected with HCoV-OC43 (100 PFU). Montelukast (10 µM) was added to the infected cells at 0, 1, 2, 4, 8, 10 h post infection. At 16 h post-incubation, the supernatant was replaced with DMEM containing 0.6% LMP agarose and 2% FBS. The plaque assay was performed and analyzed as described above.

### 2.8. Virus Attachment Assay

The HCoV-OC43 attachment experiment was performed as previously described [20,28]. HCoV-OC43 (200 PFU) were mixed with or without 10 µM montelukast in cold DMEM, then the mixture was added to BHK-21 cell monolayer immediately. After incubating for 1 h on ice, the inoculum was removed and cells were washed three times and overlayed with fresh DMEM containing 2% FBS and 0.6% LMP agarose. The plaque assay was performed and analyzed as previously described.

### 2.9. Virus Internalization Assay

For the HCoV-OC43 internalization assay, as previously described [29,30], BHK-21 cells were first infected with 250 PFU of HCoV-OC43 on ice without montelukast for 1 h. After removing the inoculum from cells and washing the cells with fresh DMEM twice, the cells were incubated with or without 10 µM of montelukast for 2 h at 33 °C. Then, the medium was removed and cells were washed twice and overlayed with fresh DMEM containing 2% FBS and 0.6% LMP agarose. The plaque assay was performed and analyzed as described above.

### 2.10. Assay to Detect Inactivated HCoV-OC43 Virions

Montelukast serially diluted in DMEM was mixed with equal volume of HCoV-OC43 (2500 PFU) as previously described [31,32]. After incubating for 2 h at 33 °C, 5 M NaCl and 50% PEG-8000 (Amresco, Solon, OH, USA) were added to the mixture at final concentrations of 0.67 M and 10% respectively, and then incubated on ice for 2 h. The mixture was centrifuged at 20,200× *g* for 1 h at 4 °C and the supernatant, containing the free montelukast, was removed and the pellet containing the HCoV-OC43 particles was washed with 3% PEG-8000 in PBS containing 10 mg/mL BSA (Amresco, Solon, OH, USA). After centrifugation again, the infectious HCoV-OC43 particles in the pellet was resuspended in DMEM and determined by plaque assay as described above.

### 2.11. RNase Digestion and RT–qPCR Assay

The RNase digestion and RT–qPCR experiments were performed to detect whether HCoV-OC43 genomic RNA was released from the virus particles after treatment of montelukast, as previously described [32,33]. Briefly, HCoV-OC43 (2000 PFU) was incubated with serially diluted montelukast for 2 h at 33 °C. Then, the released viral genomic RNA was digested by micrococcal nuclease (New England BioLabs, Ipswich, MA, USA) at 37 °C for 1 h. Then, HCoV-OC43 genomic RNA inside the intact virions was extracted using the EasyPure Viral DNA/RNA Kit (Transgen Biotech, Beijing, China). The purified viral RNA was detected by RT-qPCR using TransScript Green One-Step RT-qPCR SuperMix (Transgen Biotech, Beijing, China) and the CFX96 TM Real-Time System (Bio-Rad, Hercules, CA, USA) in accordance with the manufacturers’ instructions. The primer pair targeted HCoV-OC43 nucleoprotein gene in the viral genome: (forward) 5′-AGCAACCAGGCTGATGTCAATACC-3′ and (reverse) 5′-AGCAGACCTTCCTGAGCCTTCAAT-3′ [34].

### 2.12. Transfection Inhibition Experiment

The experiment of transfection inhibition was performed as previously described to detect the ability of montelukast to disrupt lipid envelope [35]. Briefly, the liposome-based transfection reagent Lipofectamine 2000 (Invitrogen, Carlsbad, CA, USA) was incubated with pEGFP-C1 plasmid in DMEM at room temperature for 5 min. Then, the mixture was treated with montelukast (10 µM) for 40 min, before adding the mixture to the HEK-293T cell monolayer. Twenty-four hours after transfection, the efficiency of transfection was detected by flow cytometry analysis and fluorescence microscopy.

### 2.13. Statistical Analysis

Statistical analyses were carried out using GraphPad Prism Software 7.0 (Graphpad Software Inc., San Diego, CA, USA). Probability (*p*) values were calculated by the unpaired two-tailed Student’s-*t*-test between two groups (NS, not significant; * *p* < 0.05; ** *p* < 0.01; *** *p* < 0.001; and **** *p* < 0.0001).

## 3. Results

### 3.1. Montelukast Inhibited the Infection of HCoV-OC43 in Different Host Cell Types

**Figure 1:**
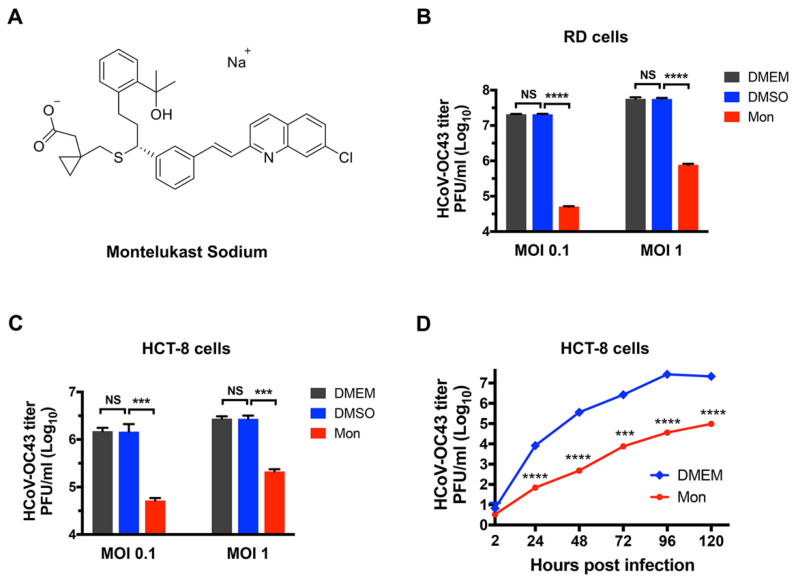
The molecular structure of montelukast (Mon) and its inhibitory activity against HCoV-OC43 in RD and HCT-8 cells. (**A**) The molecular structure of montelukast. (**B**) Montelukast inhibited the infection of HCoV-OC43 in RD cells at a multiplicity of infection (MOI) of 0.1 and 1. (**C**) Montelukast inhibited the infection of HCoV-OC43 in HCT-8 cells at MOI 0.1 and 1. (**D**) Inhibition of montelukast against HCoV-OC43 in multi-cycle replication assay. Data are represented as means ± SD. NS, not significant; *** *p* < 0.001; **** *p* < 0.0001.

First, the inhibitory activity of montelukast (10 µM) against the production of HCoV-OC43 virions was investigated in RD cells, HCT-8 human colorectal cancer cells, and MRC-5 human normal diploid lung fibroblast cells. At 48 h post-infection, the infectious HCoV-OC43 in the supernatant was harvested and determined on BHK-21 cells by plaque formation assay. Montelukast was dissolved in dimethyl sulfoxide (DMSO), and the final concentration of DMSO was no more than 0.1% in all experiments. It suggested that DMSO did not affect the production of infectious virus particle at these conditions (Figure 1B,C). In RD or HCT-8 cells at multiplicity of infection (MOI) of 0.1, approximately 2.1 × 10^7^ or 1.5 × 10^6^ progeny virions per milliliter were produced in the absence of montelukast and DMSO (Figure 1B,C), while the production of HCoV-OC43 virions, in the presence of montelukast, were reduced by 2.6 Log10 (99%) or by 1.5 Log10 (96%) in RD or HCT-8 cells, respectively, when compared with the infected cells treated with equal volumes of DMSO. Following infection at an MOI 1, it was also found that montelukast caused a significant reduction in the production of progeny HCoV-OC43 by 1.8 Log10 (98%), 1.1 Log10 (92%), or 1.2 Log10 (94%) in RD cells (Figure 1B), HCT-8 cells (Figure 1C), or MRC-5 cells (Figure S1), respectively. In order to better understand the inhibitory activity of montelukast in propagation and stepwise accumulation of the progeny viruses, the multicycle growth curves of HCoV-OC43 in HCT-8 cells with or without montelukast were analyzed. It was demonstrated that montelukast greatly reduced the titer of infectious HCoV-OC43 particles in multiple rounds of infection (Figure 1D), and suggested that montelukast has a potent and long-lasting inhibitory effect on HCoV-OC43 infection.

**Figure 2:**
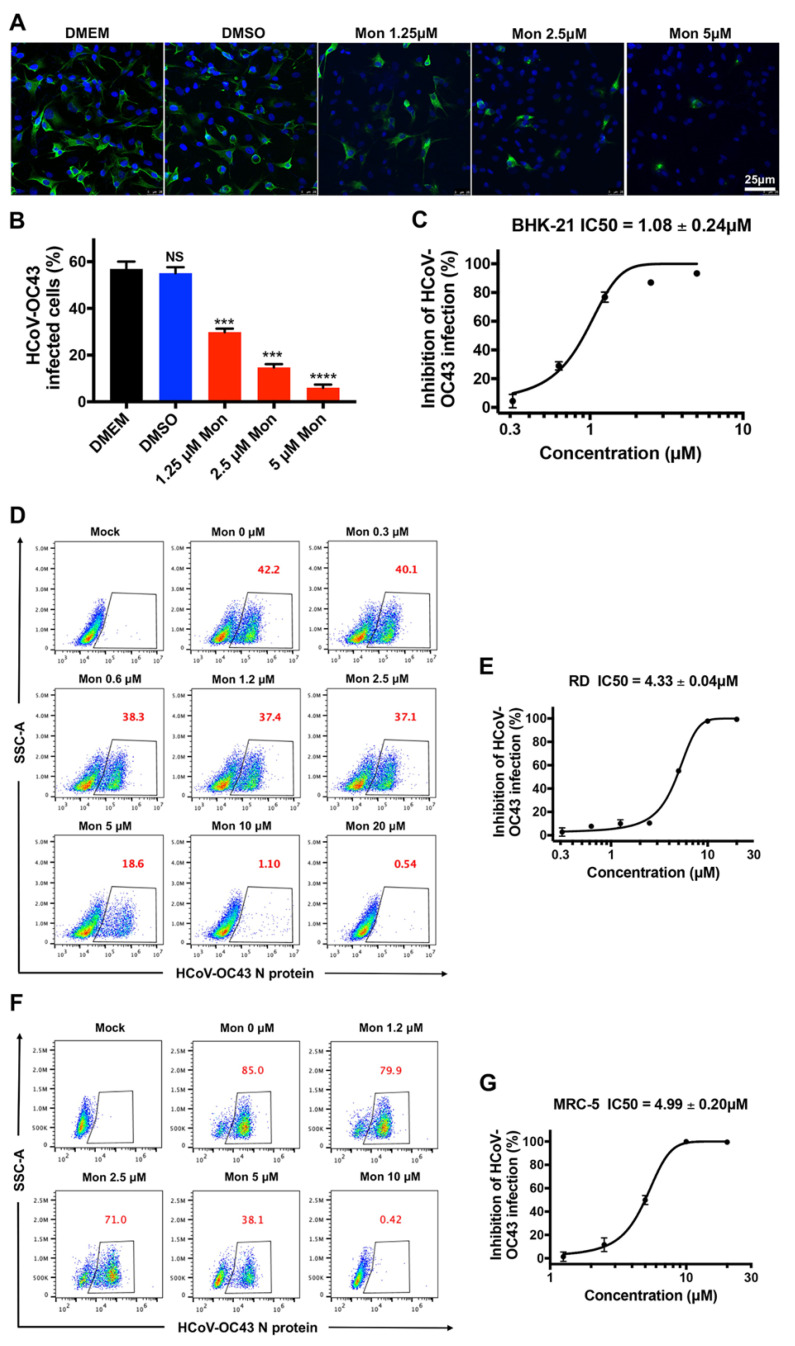
Inhibitory activity of montelukast (Mon) against HCoV-OC43 infection in a dose-dependent manner. Antiviral activity of montelukast against HCoV-OC43 in BHK-21 cells were measured by immunofluorescence assay (**A**,**B**) and in plaque reduction assay (**C**). HCoV-OC43 N protein were stained by the mouse anti-HCoV-OC43 nucleoprotein mAb (green); nuclei were stained by 4,6-diamidino-2-phenylindole (blue), scale bar: 25 µm. RD cells were infected by HCoV-OC43 with serially diluted montelukast, then the percentage of infected cells (red numbers) was evaluated by flow cytometry and the 50% inhibitory concentration (IC50) value was calculated (**D**,**E**). MRC-5 cells were infected by HCoV-OC43 with serially diluted montelukast, the percentage of infected cells (red numbers) was evaluated by flow cytometry, and the IC50 value was calculated (**F**,**G**). Data are represented as means ± SD. NS, not significant; *** *p* < 0.001; **** *p* < 0.0001.

For confirming the antiviral effect of montelukast, next we assessed the inhibitory activity of the drug on BHK-21 cells in an immunofluorescence assay by detecting nucleoprotein of HCoV-OC43 and in a plaque reduction assay. As shown in Figure 2A, treatment with montelukast reduced the number of HCoV-OC43-infected cells in a dose-dependent manner, and the viral infectivity was suppressed by nearly 50% with a concentration of 1.25 µM (Figure 2B). The plaque reduction assay corroborated these results, showing that montelukast potently inhibited infection of HCoV-OC43 with a 50% inhibitory concentration (IC50) value of 1.08 ± 0.24 µM in BHK-21 cells (Figure 2C). Furthermore, flow cytometry analysis showed that montelukast decreased the percentage of RD cells infected with HCoV-OC43 from 42.2 to 18.6%, and to 1.1%, at 5 µM and 10 µM, respectively (Figure 2D). It indicated that montelukast also had a potent inhibitory effect on the infection of HCoV-OC43 in RD cells or in MRC-5 cells with an IC50 value of 4.33 ± 0.04 µM (Figure 2E) or of 4.99 ± 0.20 µM (Figure 2G), respectively.

**Figure 3:**
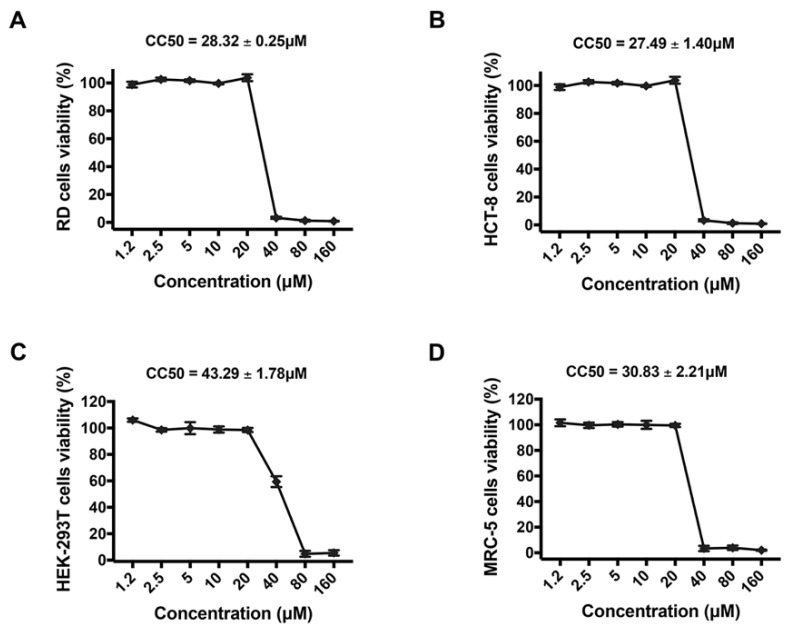
Toxicity for montelukast to different cells. (**A**) RD, (**B**) HCT-8, (**C**) HEK-293T, and (**D**) MRC-5 cells were treated with montelukast at different concentrations, and then cells’ viability was detected by Cell Counting Kit-8 (CCK-8). Data are represented as means ± SD.

In order to further verify that the potent inhibitory activity of montelukast against HCoV-OC43 infection is independent of its cytotoxicity, the viability of RD cells, HCT-8 cells, MRC-5 cells, and HEK-293T cells treated with montelukast ranging from 1.2 to 160 µM for 2 days were analyzed by CCK-8. As shown in Figure 3, montelukast did not affect the viability of the tested cells at the concentration of 20 µM or lower. All of the results demonstrated that montelukast exhibited potent antiviral activity against HCoV-OC43 infection at these concentrations without cytotoxicity in different host cells.

### 3.2. Montelukast Inhibited HCoV-OC43 Infection at Early Stage of Infection

**Figure 4:**
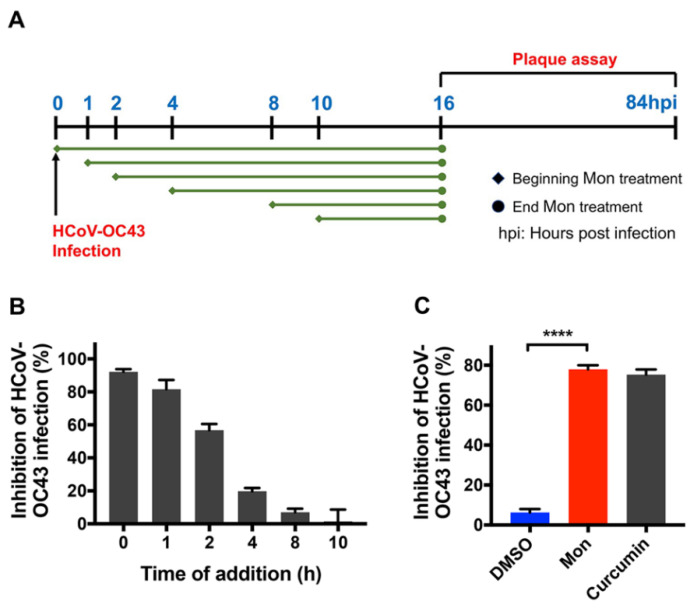
Montelukast (Mon) inhibited HCoV-OC43 infection through blockage of the early stage of the virus lifecycle. (**A**) Outline of time-of-addition experiment. hpi: hours post infection. (**B**) Time-of-addition experiment to study montelukast against HCoV-OC43 infection. Montelukast was administrated at different time point after infection and plaque assay was performed to evaluate antiviral activity of the drug. (**C**) Montelukast blocked HCoV-OC43 attachment to BHK-21 cells. The mixture of virus and montelukast was added to BHK-21 cells monolayer and incubated on ice for 1 h. Then, montelukast and the unbound virions were removed by washing. Curcumin was included as positive control. Data are presented as means ± SD. **** *p* < 0.0001.

It has been reported that some compounds and drugs inhibit coronavirus infection, targeting different stages of viral lifecycle [36]. To further investigate which step of the viral lifecycle was susceptible to montelukast treatment, a time-of-addition assay was performed (Figure 4A). Briefly, HCoV-OC43 was mixed with montelukast (10 µM) and immediately added to the BHK-21 cell confluent monolayer (time 0), or the compound was added at the indicated time points (1, 2, 4, 8, and 10 h) after viral infection. At 16 h post-infection, the supernatant was removed from the cells and the overlay medium without montelukast was added after washing the monolayer, to proceed with plaque formation experiments. As shown in Figure 4B, maximal antiviral properties were observed when montelukast was added at 0 or 1 h post-infection, and less inhibitory activity was detected when added at 2 h post-infection. However, montelukast administration at 4, 8, or 10 h after infection barely inhibited plaque formation, indicating montelukast could hardly obstruct HCoV-OC43 replication inside the cells. These results indicated it was at the early stage that montelukast suppressed HCoV-OC43 infection.

Virus infection at early stage can be further divided into two main steps: viral attachment and internalization; therefore, experiments were performed to detect the inhibitory effect of montelukast on viral attachment and internalization [20,36]. For the virus attachment assay, HCoV-OC43 were mixed with 10 µM montelukast and immediately incubated with BHK-21 cell monolayer on ice for 1 h, followed by plaque assay. As shown in Figure 4C, incubation of HCoV-OC43 with montelukast significantly reduced the number of viruses attached to BHK-21 cells, which was consistent with the result of time-of-addition experiment.

Next, we investigated whether montelukast inhibited HCoV-OC43 internalization, a post step of attachment [20,31]. BHK-21 cells were first incubated with viruses on ice for 1 h to allow only adsorption. After washing with cold DMEM to remove unbound HCoV-OC43, the temperature shifted to 33 °C to initiate virus internalization with the medium containing montelukast or chloroquine. We found that treatment with chloroquine, a known inhibitor preventing virus internalization [30], greatly decreased the number of plaques, while treatment with montelukast has no significant impact (Figure S2). These results showed that the potent antiviral activity of montelukast mainly functioned at the adsorption step of the HCoV-OC43 lifecycle, not at the internalization step.

### 3.3. Montelukast Irreversibly Inhibited HCoV-OC43 Infectivity, Due to Direct Inactivation of Virus Particles

**Figure 5:**
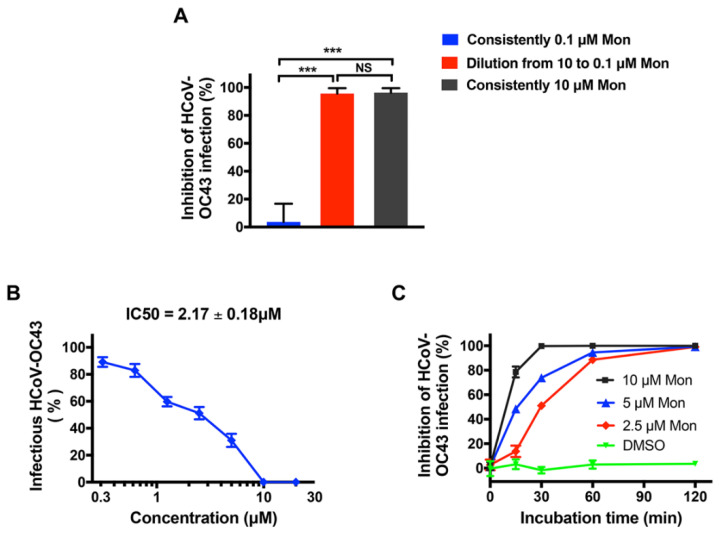
Montelukast (Mon) directly inactivated HCoV-OC43 particles in an irreversible and time-dependent manner. (**A**) Antiviral activity of montelukast against HCoV-OC43 was irreversible. The viruses were incubated with montelukast (10 µM) for 1 h, and then the mixture was diluted by 100-fold, resulting in decrease of the concentration of montelukast to 0.1 µM before performing plaque assay. (**B**) HCoV-OC43 virions were treated with serially diluted montelukast for 2 h at 33 °C. Then, viruses were precipitated and separated from montelukast by PEG-8000 and the plaque assay was performed to detect HCoV-OC43 infectivity. (**C**) Montelukast inhibited HCoV-OC43 infection in a time-dependent manner. HCoV-OC43 was treated with montelukast (2.5, 5, and 10 µM) for different times at 33 °C, and then plaque assay was performed to detect HCoV-OC43 infectivity. Data are presented as means ± SD. NS, not significant; *** *p* < 0.001.

Montelukast exerted the greatest inhibitory effects at the step of virus adsorption, therefore we wondered whether the inhibitory effect is reversible, and performed an assay to detect the reversibility of infectivity inhibition by montelukast [20,33]. Briefly, HCoV-OC43 particles were first treated with 10 µM montelukast, a concentration expected to produce about 90% antiviral activity. Then, the mixture was diluted by 100-fold, making the concentration of montelukast drop to 0.1 µM, at which montelukast had negligible impact on HCoV-OC43 infection. Meanwhile, the virus was also consistently treated with 10 or 0.1 µM montelukast before the plaque formation assay was performed. We observed that dilution from 10 to 0.1 µM could not eliminate the antiviral activity (Figure 5A), indicating that montelukast irreversibly inhibited the infection of HCoV-OC43.

The irreversible inhibition of viral infectivity by montelukast dropped a hint to us that it probably could inactivate HCoV-OC43 virions directly. Therefore, the possibility of direct inactivation of montelukast on HCoV-OC43 was evaluated as previously described [31,32]. Briefly, viruses were treated with different concentrations of montelukast, then precipitated and separated from montelukast by pelleting with PEG-8000. The number of infectious HCoV-OC43 virions were immediately determined on BHK-21 cells by plaque formation assay. As shown in Figure 5B, with the increase of montelukast concentration, the number of infectious HCoV-OC43 particles gradually decreased. Thus, montelukast could indeed directly inactivate HCoV-OC43, with IC50 value of 2.17 ± 0.18 µM.

To investigate how quickly montelukast could inactivate the virus, the mixture of HCoV-OC43 and montelukast was collected at the indicated times (15, 30, 60, and 120 min) and the plaque formation assay was immediately performed. HCoV-OC43 treated with 10 µM of montelukast almost completely lost infectivity within 30 min of incubation (Figure 5C). Additionally, as the incubation time increased, the number of infectious HCoV-OC43 virions treated with lower concentrations of montelukast gradually decreased to the same extent as with 10 µM. Overall, these results suggested that montelukast could directly inactivate virions in a dose- and time- dependent manner, hence irreversibly inhibiting HCoV-OC43 infection by blocking viral entry.

### 3.4. Montelukast Induced Release of HCoV-OC43 Genomic RNA by Disrupting the Integrity of the Virions

**Figure 6:**
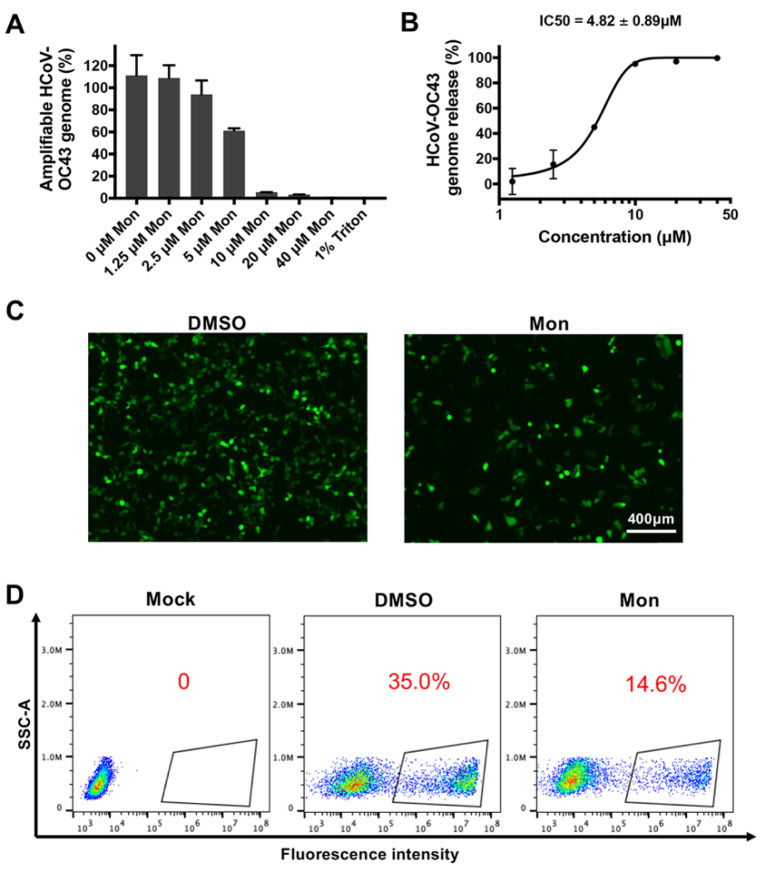
Montelukast (Mon) induced release of HCoV-OC43 genomic RNA by disrupting the integrity of the virions. (**A**). RNase digestion experiment was used to detect the genomic RNA unreleased by HCoV-OC43 treated with montelukast. The genomic RNA coding HCoV-OC43 nucleoprotein was detected. (**B**) The IC50 value of montelukast on release of HCoV-OC43 genomic RNA. (**C**) Montelukast affected the efficiency of transfection based on Lipoectamine 2000. The mixture of montelukast, EGFP plasmid, and Lipoectamine 2000 was incubated for 40 min at room temperature, and then added to the HEK-293T cell monolayer. Twenty-four hours after transfection, fluorescence microscopy and flow cytometry analysis detected the efficiency of transfection (**D**), scale bar: 400 µm; red numbers: the percentage of cells expressing EGFP. Data are presented as means ± SD.

It has been reported that certain peptides and compounds could inactivate Zika virus and release its viral genomic RNA by destructing the integrity of virus particles [31,32]. By employing the method of viral RNA digestion as described previously, we found that the RNA genome of intact HCoV-OC43 particles would be protected from digestion of micrococcal nuclease. However, the genomic RNA of HCoV-OC43, whenever treated with montelukast, would be digested in different degrees depending on montelukast’s concentration correspondingly (Figure 6A). So, this indicated that montelukast could induce the release of HCoV-OC43 genomic RNA by disrupting the integrity of the virions, with the IC50 value of 4.82 ± 0.89 µM (Figure 6B).

The ability of montelukast to release viral RNA of both HCoV-OC43, discovered in this study, and Zika virus, reported previously [20], implied that the target of montelukast may be the same component in the viral shell that both the two viruses possess, which most likely be the lipid bilayer membrane encapsulating the viral genomes. Then, it was tested by employing a liposome-based transfection reagent, Lipofectamine 2000, which has the lipid structure to mimic the viral envelope [35]. Briefly, Lipofectamine 2000 and the plasmid pEGFP-C1 expressing enhanced green fluorescence protein (EGFP) were diluted together in DMEM, followed by addition of montelukast and incubation for 40 min at room temperature. The efficiency of transfection on HEK-293T cells was determined subsequently. As shown in Figure 6C,D, adding montelukast to the mixture of Lipofectamine 2000 and plasmid decreased the number of cells expressing EGFP by more than half. These results indicated that montelukast destroyed the liposome and reduced the efficiency of transfection, suggesting that montelukast could probably disrupt the lipid membrane of the HCoV-OC43, then directly inactivate virus particles.

By employing propidium iodide (PI), a red-fluorescent membrane-impermeable dye which is excluded from cells with intact membranes, while penetrates damaged cell membranes to stain DNA in the nuclei, we found that montelukast slightly damaged the plasma membrane of RD cells at high concentration (20 µM), and the cell membranes remained intact at the concentration of no more than 10 µM (Figure S3). The digitonin, a compound usually used to increase cellular permeability as positive control, obviously damaged cell membrane at 5 µM. The different susceptibility between cells and HCoV-OC43 to montelukast may reflect the discrepancy between cellular membrane and viral membrane, which is derived from membrane of the endoplasmic reticulum–Golgi intermediate compartment (ERGIC), where assembly and budding of coronavirus occurs [37].

## 4. Discussion

HCoV-OC43, the prototype member of the genus beta-coronavirus which likely originated from rodents [38,39], is not only responsible for 15–30% of cases of common colds in humans each year [30,40] but is also able to cause pneumonia and viral encephalitis [41,42,43]. More attention should be paid to therapies for diseases caused by HCoV-OC43. However, there is no vaccine or direct-acting antiviral agent available for the prevention and treatment of infection of HCoV-OC43 at present.

Repurposing of Food and Drug Administration (FDA)-approved drugs may be an effective method to discover antiviral agents. In this way, montelukast was founded as a potent inhibitor against the infection of HCoV-OC43. As a leukotriene receptor antagonist, montelukast has been approved for the treatment of asthma patients for many years [44]. In the study, it was shown that montelukast caused a significant reduction in the production of progeny infectious HCoV-OC43 particles (Figure 1B,C and Figure S1), not only at multiple of infection (MOI) 0.1 but also at MOI 1. Additionally, we also found that montelukast potently inhibited HCoV-OC43 infection in a dose- and time-dependent manner (Figure 2 and Figure 5C). The viral infectivity was suppressed by nearly 50% with the treatment of 1 µM of montelukast, and the HCoV-OC43 almost completely lost infectivity when incubated with 10 µM of montelukast for 30 min. These results show that montelukast was an effective inhibitor against the infection of HCoV-OC43 in vitro. Moreover, the results of further research suggested that montelukast could disrupt the integrity of the HCoV-OC43 virions, resulting in the release of the viral genomic RNA from the interior of the virus treated with montelukast (Figure 6A,B). Recently, our team reported that montelukast exhibited potent antiviral activities against ZIKV, DENV-2, and YFV 17D by inducing the release of viral genome RNA and hence disrupting the integrity of these viruses, while the integrity of non-enveloped virus enterovirus 71 (EV-71) was not affected [20]. Interestingly, the property shared by both HCoV-OC43 and the viruses mentioned above, except for EV-71, is that they are all enveloped viruses coated with lipid membrane. This gave us a hint that the target of montelukast might be the lipid envelope of virus particles, which was corroborated by DNA transfection experiments with Lipofectamine 2000, a liposome-based reagent (Figure 6C,D).

SARS-CoV, SARS-CoV-2, and MERS-CoV are other highly pathogenic members of beta-coronavirus with high morbidity and mortality. Although SARS-CoV and MERS-CoV have disappeared temporarily after the outbreak, it is important to keep a close watch on the risk of re-emerging. COVID-19, the disease caused by a novel coronavirus SARS-CoV-2, has resulted in approximately 318 million confirmed cases and more than 5.5 million deaths until now, presenting an urgent need and an unprecedented challenge to explore effective vaccines and drugs for prevention and treatment [14]. Continuous mutations of the S protein have rendered resistance to vaccine sera or many monoclonal antibodies [12]. Unfortunately, only a few small molecule drugs, such as remdesivir and molnupiravir, are conditionally approved in limited countries and regions, which target the replication of SARS-CoV-2 inside the cells. The latest study by Serdar Durdagi et al. suggested that montelukast, with high concentrations, blocked SARS-CoV-2 pseudovirus entry into the host cell (EC50: more than 40 µM) and inhibited the activity of main protease enzyme (IC50: 28.36 µM) [45]. The homology of Mpro between HCoV-OC43 and SARS-CoV-2 is about 48% and the IC50 (~28 µM) of montelukast against SARS-CoV-2′s Mpro reported by them is much higher than the concentration we used, which may explain why only entry of HCoV-OC43 was affected in this study. It was also found that montelukast sodium hydrate (MSH) significantly inhibited the entry of MERS-spike pseudovirion at 20 µM, indicated by an enhanced reduction in luciferase activity, and IC50 values of MSH with authentic MERS-CoV in both pre-treatment and co-treatment experiments being both approximately 3 µM [17].Due to the shortage of biosafety level three containment facilities to us, the inhibitory effect of the montelukast on authentic SARS-CoV-2 virus entry could not be tested at present. However, considering the similarity between SARS-CoV-2 and HCoV-OC43, it is likely that montelukast might block SARS-CoV-2 entry in the same way as montelukast blocks HCoV-OC43.

The dosage recommended for adults and adolescents 15 years of age and older is 10 mg daily by oral administration. The maximum plasma concentration of montelukast after oral administration of 10 mg ranged from 0.58 to 0.63 µM in healthy female and male subjects, respectively [46], which is lower than the IC50 of montelukast on HCoV-OC43 inhibition in this study. It has been reported that a formulated montelukast nasal spray containing 0.5–15 µg/50 µL (16–493 µM) of montelukast, which is much higher than the IC50 of montelukast on HCoV-OC43 inhibition, is ideal for nasal administration for treatment of allergic rhinitis, and has no toxicity nor results in alteration of the tight junction of nasal epithelial cells [47]. Hence, the preferable route of administration for clinical use of montelukast or montelukast-like compound against airborne beta-CoV may be nasal spray to obtain local delivery with effective concentration.

The main limitation of this study is that the direct observation of montelukast-disrupted virosomes or, even better, the HCoV-OC43 virions, was hampered by the limited availability of the electron microscopy facility during the pandemic. The transmission electron microscopy is widely used for analysis of the morphology of viruses, including SARS-CoV-2 [48,49], ZIKV [50], and influenza virus [51], which will contribute to visualization of the damaged HCoV-OC43 virions caused by treatment of montelukast. In infected cells, coronaviruses induce the formation of double-membrane vesicles (DMVs), in which the viral replication–transcription complexes (RTCs) are located. It is interesting as to whether montelukast could damage these vesicles, although the apparent permeability of montelukast is relatively low and the cellular accumulation of montelukast ranges from 20 to 60 pmole/3 × 10^6^ cells/h [52], indicating that it is unlikely that the DMVs would be disrupted by montelukast. To validate this, transmission electron microscopy or cryo-electron microscopy combined with electron tomography could be adopted, which have been recently employed to visualize the structure of DMVs induced by coronavirus infection [53,54,55]. Alternatively, DMVs could also be isolated for analysis [56,57].

To conclude, montelukast, used safely for over 20 years in asthmatic patients, behaves similar to an entry inhibitor against infection of HCoV-OC43 in a dose- and time- dependent manner, which directly and irreversibly inactivates HCoV-OC43, probably by targeting the viral lipid envelope, and the ability and detailed mechanism of montelukast for inactivating and blocking entry of other beta-coronaviruses is worthy of further investigation.

## Data Availability

The data presented in this study are available on request from the corresponding author.

## Supplementary Materials

The following supporting information can be downloaded at: https://www.mdpi.com/article/10.3390/v14050861/s1, Figure S1: Montelukast (Mon) inhibited the infection of HCoV-OC43 in MRC-5 cells at multiplicity of infection (MOI) of 1; Figure S2: HCoV-OC43 internalization assay; Figure S3: Propidium iodide (PI) staining of RD cells treated with montelukast (Mon) or digitonin.
