# Supplementary material for: Montelukast Inhibits HCoV-OC43 Infection as a Viral Inactivator
Source: Viruses. 2022 Apr 21;14(5):861. doi: 10.3390/v14050861 (PMC9143845; doi:10.3390/v14050861)
Supplement: Supplementary file 1 [file viruses-14-00861-s001.zip › viruses-1598309-supplementary.pdf]

viruses

Supplementary Materials

Montelukast Inhibits HCoV-OC43 Infection as a Viral Inactivator

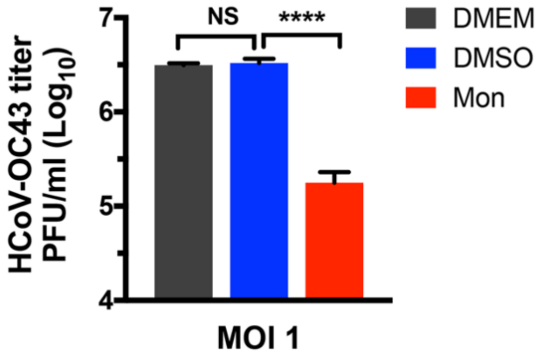

Figure S1. Montelukast (Mon) inhibited the infection of HCoV-OC43 in MRC-5 cells at multiplicity of infection (MOI) of 1**.** MRC-5 cells (2×10^5) were infected with 2×10^5 PFU of HCoV-OC43 after viruses were treated with or without 10 μM of montelukast for 1 h at 33°C. Then the inoculum was removed from cells after 2 h incubation at 33 °C and fresh medium containing 2% FBS and supplemented with or without 10 μM of montelukast was respectively added. The supernatant was collected at 3 days after infection, and the infectious HCoV-OC43 particles were determined by plaque formation assay. Data are presented as means ±SD. NS, not significant; **** $p < 0.0001$.

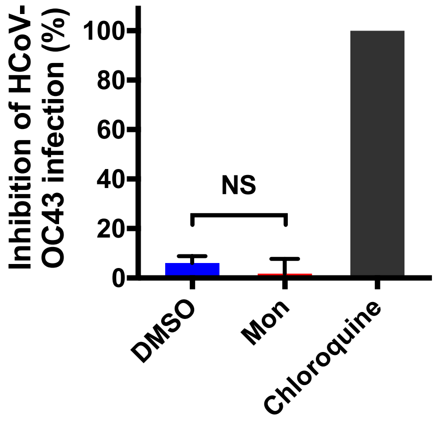

Figure S2. HCoV-OC43 internalization assay. BHK-21 cells were first infected with 250 PFU of HCoV-OC43 on ice for 1h. After removing the inoculum from cells and washing the cells with fresh DMEM twice, the cells were incubated with or without 10 μM of montelukast for 2 h at 33°C to allow virus internalization. Then the plaque assay was performed. Chloroquine was included as positive control. Data are presented as means ±SD. NS, not significant.

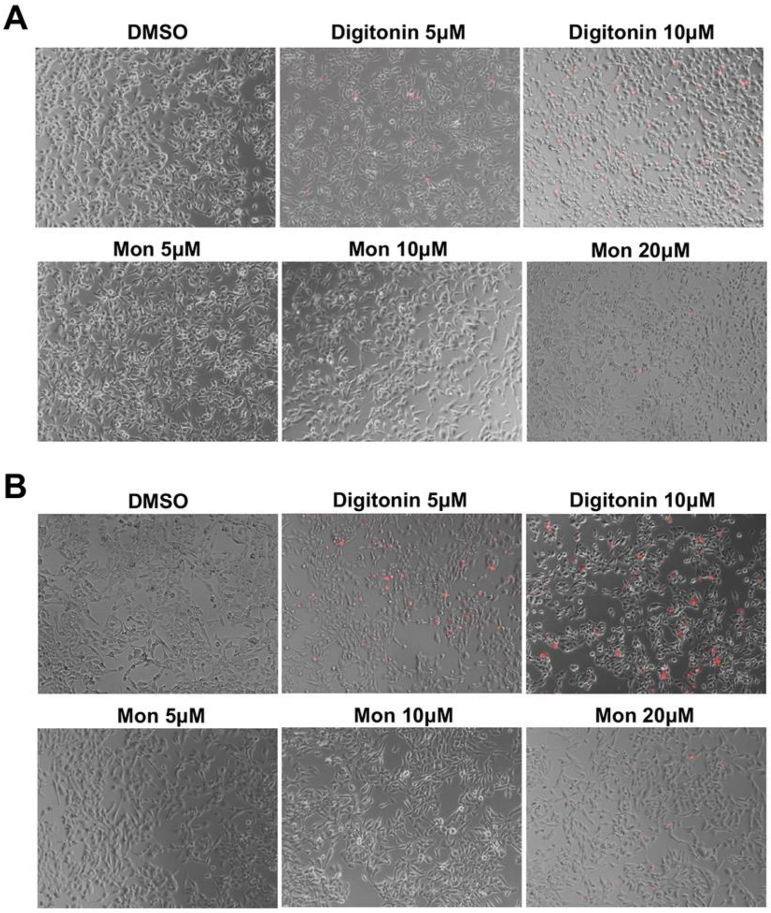

Figure S3. Propidium iodide (PI) staining of RD cells treated with montelukast (Mon) or digitonin. RD cells were seeded in cell culture plates and incubated overnight at 37°C. Cells were treated with Mon (5, 10 and 20 μM) or digitonin (5 and 10 μM) respectively for 2 h **(A)** or 6 h **(B)** at 37°C. After removing the supernatant and washing cells twice with fresh DMEM, 10 μg/ml of PI in DMEM was supplemented for 20 min incubation. Then the cells were observed for PI staining (red puncta) with a fluorescence microscope (100×).
